# An innovative approach to screening and chemoprophylaxis among contacts of leprosy patients in low endemic settings: experiences from Cambodia

**DOI:** 10.1371/journal.pntd.0007039

**Published:** 2019-03-28

**Authors:** Arielle Cavaliero, Helena Greter, Thomas Fürst, Sambath Lay, Sarady Sao Ay, Jan Robijn, Peter Steinmann

**Affiliations:** 1 Novartis Foundation, Basel, Switzerland; 2 Swiss Tropical and Public Health Institute, Basel, Switzerland; 3 University of Basel, Basel, Switzerland; 4 National Leprosy Elimination Program, Phnom Penh, Cambodia; 5 Netherlands Leprosy Relief, Hanoi, Vietnam; University of Tennessee, UNITED STATES

## The challenge

Leprosy is endemic in numerous poverty-affected communities. Many years usually pass between an infection with *Mycobacterium leprae* and the onset of clinical signs of leprosy. The inconspicuous first symptoms and slow progress of the disease often result in considerable delay until the diagnosis is established and treatment initiated [1]. Contributing to this delay are the generally weak health systems in areas where leprosy is endemic. Additionally, health personnel are often unfamiliar with the cardinal signs of leprosy, which can lead to misdiagnoses [2].

The introduction of multi-drug therapy (MDT) in the 1980s has resulted in a massive reduction of the number of registered leprosy patients [3, 4]. MDT drugs were first donated by the Nippon Foundation (1995–1999) and since 2000 by Novartis. Following the global achievement of “leprosy elimination as a public health problem” in 2000, the efforts to actively identify leprosy patients have been drastically scaled back in most countries. Instead, passive case detection and integration of leprosy services into the general health system have become standard [5]. Unfortunately, achieving elimination as a public health problem has not halted leprosy transmission [4, 5].

Close and long-term contact with an untreated leprosy patient is considered to be a major risk factor for infection [6]. The administration of a single dose of rifampicin (SDR) as postexposure prophylaxis (PEP) to leprosy negative contacts of recently diagnosed patients has been shown to reduce their risk of developing leprosy over the following years [7, 8]. The feasibility and impact of a combination of contact tracing, screening, and PEP with SDR is currently being evaluated in eight countries in the frame of the Leprosy Post-Exposure Prophylaxis (LPEP) program [9, 10].

Cambodia declared leprosy elimination as a public health problem in 1998. Following this milestone, leprosy control was largely halted, and the National Leprosy Elimination Program (NLEP) atrophied. One decade later, a build-up of undiagnosed leprosy patients was observed, and the concept of retrospective active case finding (RACF) campaigns or “drives” was developed. At its core, a drive is carried out by a mobile team of specifically trained leprosy experts who systematically trace the leprosy patients diagnosed in an operational district (OD) and screen their contacts. From 2011 until 2015, the contacts of all leprosy patients diagnosed across Cambodia between 2001 and 2010 were screened in the frame of 11 drives [11]. Recognizing the mutually complementing nature of routine passive case detection and RACF, as well as interest in exploring the potential of PEP with SDR, the NLEP decided to pilot an enhanced scheme in which SDR PEP is integrated into the drives. The objective is to evaluate operational feasibility in a low-endemic setting to (a) maintain or revitalize contact tracing by RACF and (b) integrate SDR PEP into RACF.

## The solution

First, priority ODs for implementation of the project were identified by analyzing data for the years 2011 through 2015 from the national leprosy database. A relatively high number of new leprosy patients per 100,000 total population diagnosed in an OD over the period 2011 through 2015 was defined as the most important selection criterion. This is justified by the aim of the project, namely, the screening of contacts of known recent patients to improve early diagnosis and prompt treatment among this high risk group. By default, the top 25% of the ranked OD list were selected as priority ODs. Additionally, three secondary criteria were defined: (i) as an indication for the level of transmission: the proportion of children <15 years among all leprosy patients in an OD, in which priority was given to the top quartile (25%) of the values; (ii) as an indication for the level of diagnosis delay: the proportion of multibacillary (MB) cases among all patients in an OD (top quartile); and (iii) as an indication for the level of stigmatization: the proportion of women among all patients in an OD (bottom quartile). At least two out of three secondary criteria needed to be fulfilled for the inclusion of an OD into the list of priority ODs.

Applying these criteria, 31 out of all 78 ODs recognized at the time of the analysis were selected (Table 1)

**Table 1:** Number of ODs prioritized for inclusion in the RACF with SDR PEP project in Cambodia, stratified by selection criteria.

**Fulfilled inclusion criteria**	**Number of ODs**
Primary criterion^1^	20
Secondary criterion (subcriteria 2a^2^, 2b^3^, and 2c^4^)	1
Secondary criterion (subcriteria 2a and 2b only)	1
Secondary criterion (subcriteria 2a and 2c only)	1
Secondary criterion (subcriteria 2b and 2c only)	8
Total	31

^1^high number of new cases per 100,000 population.

^2^high proportion of child cases.

^3^high proportion of MB cases.

^4^low proportion of female cases.

**Abbreviations:** MB, multibacillary; OD, operational district; PEP, postexposure prophylaxis; RACF, retrospective active case finding; SDR, single dose of rifampicin.

All leprosy patients (index patients) newly diagnosed in the priority ODs since 2011 and still residing in a priority OD at the time of the drive are eligible for participation. The exact number of resident index patients is determined in the frame of a predrive visit to the OD. The contacts targeted in the frame of the project include household members of the index patients, defined as individuals living in the same household as the index patient for at least 3 months prior to the visit of the drive team, and the neighbors who are residing in the five households located closest to the index patient’s household. To strengthen awareness and avoid absenteeism, the index patients and their contacts are informed of the drive and the exact time of the home visit a few days prior to the drive. Upon arrival of the drive team in a household, the leprosy patient is briefed about the aims, procedures, benefits, and potential risks of the intervention, and written informed consent to disclose the disease status to all household members and neighbors is sought. Upon signature, a list of all household members and neighbor contacts is established. All listed individuals are informed about the program, procedures, the possible consequences of the different screening outcomes (confirmed leprosy, suspect leprosy, leprosy negative, which means the contact is potentially eligible for PEP with SDR) as well as on the risks and benefits related to SDR administration. Contacts are then asked to provide informed consent to participate in screening and SDR PEP, only the former, or withdraw from the study.

A competent clinician embedded into the drive team can directly confirm leprosy diagnoses, upon which newly diagnosed patients are registered and start MDT as per the routine NLEP guidelines. Screening-negative contacts are assessed for SDR eligibility against a list of exclusion criteria and receive SDR at the appropriate dose [9]. Eligible contacts residing in a household in which a new leprosy patient has been detected during the drive receive SDR only one month after the start of MDT of the new patient. For this follow-up, a health center or district staff returns to the household to reassess eligibility and administer the correct dose. An individual SDR card with the contact identification, date, and rifampicin dose is handed out to each participating contact. Last, SDR recipients are advised to seek care at the nearest health facility should they develop any symptoms other than the known side effects of rifampicin such as discoloration of urine.

The existing data collection and reporting system of the Cambodian NLEP is used to plan the drives, and only drive-specific data—notably, contact details, informed consent, screening and SDR administration—are recorded on separate forms. The drive data are entered into a project database and integrated into the NLEP registry.

## Outlook

The National Ethics Committee for Health Research of the Ministry of Health, Cambodia, has approved the protocol on 26 August 2016. Following approval and a preparation phase, three drives covering index patients living in 4 ODs had been implemented before the first interim review of the project. In the frame of these drives, a total of 86 index patients had been traced and 855 contacts consented to participate in screening. Among them, four new leprosy patients were diagnosed and 828 contacts received SDR whereas 23 eligible contacts refused it (2.7%). Another 508 contacts were absent at the time of the screening. Of note, absenteeism decreased from 43.7% of the eligible contacts during the first drive to 26.2% during the third drive. This improvement was achieved through more precise information of the index patients and their contacts on the upcoming drive, and the arrangement of fixed appointments for the passage of the drive team. Based on the emerging drives database, the feasibility of combining RACF with SDR administration will be assessed in terms of coverage, screening participation, and SDR refusal. The impact will be measured by monitoring the number and characteristics of new leprosy patients identified through contact screening, the temporal evolution of the number of new leprosy patients in the intervention areas, and the incidence of leprosy among the contacts who had received SDR.

Adjustments will be necessary to make the approach relevant for other areas with low and persisting leprosy endemicity, depending on the local health system organization and capacity. Further operational research is also required to identify acceptable approaches to contact screening and SDR administration in urban areas, in mobile populations, and in areas experiencing a high level of stigma. In addition, the most appropriate contact definition and operational parameters remain to be determined for different settings.

More generally, the in-depth analysis of the data emerging from this project in Cambodia will provide evidence on the feasibility and impact of the approach in low-resource and low-endemicity areas, complementing the emerging evidence from the LPEP program [12]. Such evidence is critical because over time, even more leprosy control programs will face a combination of low endemicity, reduced prominence, and a need to justify the costs of their work by selecting the most efficient interventions to achieve leprosy morbidity control and—ultimately—transmission interruption.
